# High Challenge Exercise and Learning Safe Landing Strategies among Community-Dwelling Older Adults: A Randomized Controlled Trial

**DOI:** 10.3390/ijerph19127370

**Published:** 2022-06-16

**Authors:** Marina Arkkukangas, Karin Strömqvist Bååthe, Anna Ekholm, Michail Tonkonogi

**Affiliations:** 1 Department of Medicine, Sport and Fitness Science, School of Education, Health and Social Studies, Dalarna University, 791 88 Falun, Sweden; ksb@du.se (K.S.B.); mtn@du.se (M.T.); 2 Department of Physiotherapy, School of Health, Care and Social Welfare, Mälardalen University, 721 23 Vasteras, Sweden; 3 Research and Development in Sörmland, Region Sörmland, 632 17 Eskilstuna, Sweden; anna.ekholm@regionsormland.se

**Keywords:** exercise, judo, martial arts, motor skill, physical activity, older adults

## Abstract

There is limited research on optimal exercise programs that effectively decrease falls and fall-related injuries in older populations. This randomized controlled trial (RCT) aimed to explore the effects of a 12-week Judo4Balance program on falling techniques, physical and psychological functions, health status, and physical activity levels among 200 community-dwelling older adults (79% women and 21% men) with a mean age of 72 years. The 200 participants were randomly allocated for the Judo4Balce program (*n* = 100) or control group (*n* = 100). The RCT intervention started in mid-January 2020 and was abruptly interrupted because of the COVID-19 pandemic. A restart of the RCT was initiated in September 2021, and the 12-week intervention was offered to two groups. This study reports the results from three points of assessment: baseline, 20-month follow-up, and 12-week postintervention. At 20 months follow-up, the control group had significantly decreased physical activity levels (summer *p* = 0.002 and winter *p* = 0.003); similar changes were not seen in the exercise group. In the exercise group, learning falling techniques in 6–9 weeks led to sustained fall competence at 20 months follow-up. Further, significant improvements in physical function (exercise group *p* = 0.009 and control group *p* < 0.001) and learning falling techniques (*p* < 0.001 for both groups) were noted in both groups after the 12-week intervention. This effective, supervised, group-based, high-challenge multicomponent exercise program needs to be further evaluated for possible impact on falls and fall-related injuries.

## 1. Introduction

With aging, a decrease in physical functioning is common, and one of its consequences is an increased risk of falls. However, there is extensive evidence that falls, to a large extent, are preventable [1]. Falls are the second leading cause of death from unintentional injuries worldwide, with the highest prevalence in adults aged ≥60 years [1]; as such, appropriate actions need to be addressed. Many risk factors for falls have been identified; of particular importance are lower limb muscle strength, gait, and balance, all of which can be improved with appropriate exercise [2,3]. Evidence has demonstrated that many falls are preventable through regular strength and balance exercise programs [4,5,6,7]. It is recommended that these programs be designed with progressively challenging exercises at a sufficient dose to maximize their benefits in reducing falls [8]. In clinical practice and research, programs and/or prescribed fall prevention exercises may be less intense, not delivered as envisaged, and monitoring of fidelity “in the field” may be lower quality or non-existent [9,10]. Participation in and adherence to fall prevention are crucial, and older adults have expressed that social aspects, time spent exercising, professional support, and individually tailored exercise programs are important to promote acceptance and participation in fall prevention exercises [11,12]. So as to address the social context, group-based fall prevention exercises have shown to be effective, especially when supervised by a healthcare professional [7,13].

One important aspect of fall prevention programs is how to address fall-related injuries and fractures; however, evidence for effective programs is lacking. One overlooked component of fall prevention is learning safe landing strategies to reduce fall-related injuries and fractures. Learning how to get down and up from the floor has been used to some extent [7,14], and learning appropriate falling techniques has been sparsely investigated. Therefore, the interest in learning falling techniques and whether these skills can be learned and performed by older adults and serve as another solution to reduce fall-related injuries is of great interest for further exploration. There is evidence for exercise targeting specific motor skills to regain stability to avoid falls using reactive stepping and power training [15,16]. Practicing a specific motor skill to respond to a trip or slip and thus regaining stability has also been suggested to reduce fall prevalence. Motor skill is defined as the ability to perform a predetermined movement pattern with maximum certainty [17].

In summary, falls remain a major threat to older adults’ health, with a continued increase in prevalence [1], and optimal designs of effective fall prevention programs need to be explored further to change the trend. Thus, the challenge remains in designing effective programs for maximal uptake, as well as promoting participation in such programs for older adults to prevent falls and fall-related injuries from a long-term perspective. Therefore, the aim of this study was to investigate a newly developed exercise program, Judo4Balance, which is a group-based, multicomponent, high-challenge exercise program that includes teaching falling techniques. The program has previously been pilot-tested for feasibility in a small sample of older adults [18]. The overall aim of this study was to explore the effects of a 12-week Judo4Balance program on falling techniques, physical and psychological functions, health status, and physical activity levels among community-dwelling older adults.

## 2. Materials and Methods

### 2.1. Study Design and Population

Participants were recruited in this randomized controlled trial (RCT) through social media (Facebook) and direct contact with retirement organizations, where the study was presented at a number of local meetings. The inclusion criteria were as follows: age > 65 years, ability to understand written and verbal Swedish language, and ability to walk independently. Exclusion criteria included uncontrollable high blood pressure or retinal detachment that did not allow for exercise.

A power calculation based on the Short Physical Performance Battery, SPPB, the main outcome variable, was performed before the start of the study. An estimated small meaningful change was set to 1 point, with an SD of 2.5, in the SPPB. This resulted in the need for 100 participants in each group (a total of 200), with a significance level of 5% and a power of 80%. For calculations, we used one-way analysis of variance (PASS 13 Power Analysis and Sample Size Software (2014), NCSS, LLC., Kaysville, UT, USA).

The first 200 participants who showed interest in the study were contacted by phone and provided information about the study by four research assistants in the research group. If older adults were willing to participate, they were contacted by three test leaders for baseline measurements. If the participants met the study inclusion criteria, informed consent was obtained. A random allocation sequence was performed based on the two groups and was transferred to consecutively numbered envelopes that were handled by one of the researchers in the research team who did not participate in data collection or intervention. When restarting the study after 20 months because of the coronavirus disease (COVID-19) pandemic, the research assistants contacted the participants for follow-up measures. After the follow-up tests, a 12-week exercise intervention was offered to both groups, if consent was required.

**Figure 1:**
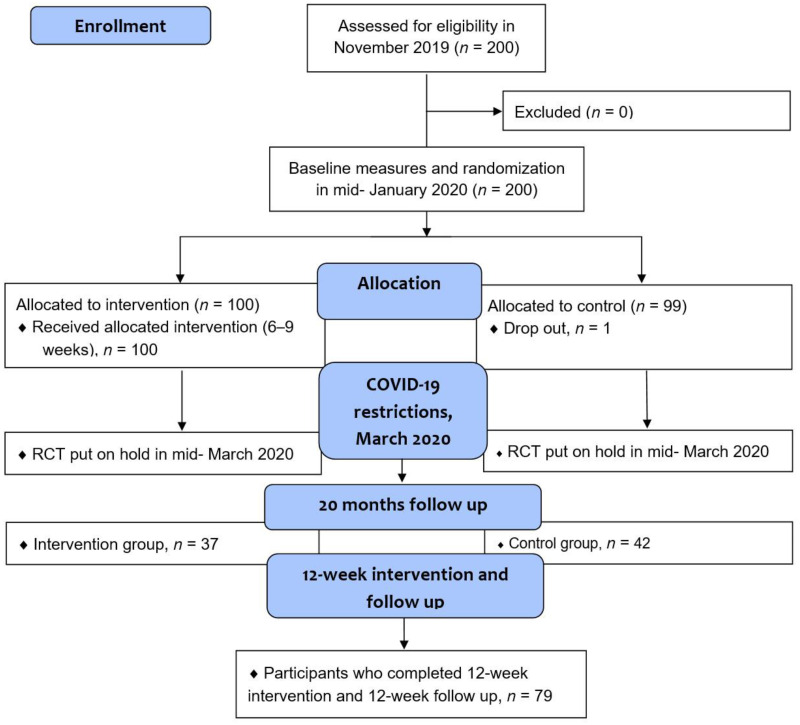
Flowchart.

All the first 200 participants who showed an interest in the study participated. The training was conducted at nine different sites in Sweden, where the judo clubs were located. Participants at each training site across different regions of the country in large, medium, and small cities were consecutively randomized into either an exercise or control group. The test leaders who performed the baseline tests were blinded to the group to which the participants were allocated. The test leaders were familiar with the tests included in the study, and all were licensed Judo4Balance instructors. The same procedure was performed at the same locations when the study restarted at the 20-month follow-up. See the flowchart in Figure 1.

### 2.2. Intervention

The intervention was a standardized multicomponent Judo4Balance exercise program, which consisted of a weekly 50–60-min group exercise program led by two licensed Judo4Balance instructors at each site. The program consisted of three main blocks over a 12-week period:

(1) Practicing fall techniques and strength exercises, body awareness, mobility training, building up load of resistance in muscles, tendons, joints, and skeleton, as well as highly challenging exercises to train balance by performing movements that are not usually carried out in everyday life activities, e.g., getting up and down from the floor;
(2) Continuing fall techniques and strength exercises, increasing load in strength exercises, challenging balance and coordination ability, greater range of movements in exercises, possibly power in exercises, continuing to build up load resistance in muscles, tendons, joints, and skeleton;
(3) Training in ability to develop power (fast power), power in strength exercises, and possibly in training in fall techniques, challenging one’s balance with increased difficulty.

The Judo4Balance is a structured and standardized program, and all judo instructors were well acquainted with the program as well as experienced in performing group exercises. Each training session has a similar pattern, starting with a warm-up, proprioception and breakfall techniques, strength training, and cool down and relaxation. Many of the exercises are performed in pairs (a common practice in judo).

Some of the exercises were performed in pairs to enable the participants to support and challenge one another through different exercises. Following the findings of a feasibility study on the Judo4Balance program in 2018 [18], the intervention was designed as a 12-week program with progressive exercise challenges.

### 2.3. Measurements

Outcome measures in the study were physical performance using the short physical performance battery (SPPB) scale, falling techniques in three directions, Mini-BESTest and Falls Self-Efficacy Scale Swedish version (FES-S), activity level by Frändin/Grimby, and self-rated health using the Euro Quality of Life Visual Analog (EQVAS) scale.

Physical performance was measured using the SPPB scale, which evaluates the functioning of the lower extremities (i.e., standing balance, gait speed, and repetitive chair stand) in older individuals [19]. The tasks were graded on a 4-point scale, with a maximum score of 12 points, where the maximum value indicates the best physical performance.

Falling techniques were measured using Strömqvist Bååthe Falling Technique Tests [20].

Falling backward: The person is asked to lay on his/her back on the mat, lift his/her head up from the mat, and stand up again. If successful, he/she scores 1 point. The next step is to sit up on the mat on one’s buttocks and fall backward. If successful, he/she scores 2 points. The subsequent step is to fall backward safely from a squatting position (3 points), and the final step is to fall safely from a standing position, which results in 4 points if performed correctly.

Falling forward: A similar progression is made for the forward breakfall strategies: laying on the stomach, falling from the knees, squatting, and falling from a standing position (forward rolling or “cat breakfall”) without any harmful maneuvers (4 points).

Falling sideways: This test was built using the same methodology. Laying down and rolling to one’s side and standing up scored 1 point, falling from sitting on the buttocks to the side scored 2 points, falling to the side from the knees or squatting scored 3 points, and finally, falling sideways from a standing position without any harmful maneuvers scored 4 points.

Balance was measured using the Mini-BESTest, which includes 14 different tasks with the following four subscales: anticipatory, reactive postural control, sensory orientation, and dynamic gait [21]. All tasks were graded from 0 to 2 points, with a maximum total score of 28 points.

To measure confidence in a person’s ability to perform various daily activities without falling, the FES-S was used. The scale includes 13 items, with a total score of 130. The scale score ranges from 0 to 10, with 0 representing low fall self-efficacy and 10 representing high fall self-efficacy [22].

Activity level was registered at baseline to describe the participants’ activity habits from the start of the study. In addition, activity level was evaluated postintervention using the Frändin/Grimby activity, where activity level was estimated on a 6-point scale [23]. The scale considers activities typically performed during the winter and summer, with higher scores representing higher activity levels.

The EuroQoL-5D-3L and EQVAS were used to measure the quality of life and self-rated health on a vertical visual analog scale (VAS). The scale was labeled “the best health you can imagine” and “the worst health you can imagine”, with a score ranging from 0 to 100. The VAS is a quantitative measure of health outcomes and reflects a patient’s judgment [24].

### 2.4. Data Analysis

For descriptive statistics, the mean, standard deviation (SD), median, minimum, and maximum were used. Nonparametric methods were used to perform all tests since the outcomes were ordinal data. Differences between the exercise and control groups were tested using the Mann–Whitney U test (two independent samples).

The Wilcoxon signed-rank test (two related samples) was used to test the difference between the baseline and values at the two follow-ups. Two-tailed *p*-values were used, with a critical significance level of 0.05. All analyses were performed using the statistical program SPSS 22.0 for Windows (IBM Corp., Armonk, NY, USA).

## 3. Results

Initially, 200 participants were included in the study (one dropout from the control group), as shown in Table 1, and there were no significant differences between the groups at the first baseline assessment. The RCT was put on hold in March 2020, and the intervention was ongoing for 6–9 weeks before abruption due to the COVID-19 pandemic. Since the study was on hold, a decision was made by the research team in September 2021 to start the intervention by offering both groups exercise intervention.

Follow-up was performed 20 months after the baseline measurement with a total of 79 participants available for the follow-up and restart of the study: 37 from the Judo4Balance group and 42 from the control group. The follow-up showed that the control group had significantly decreased activity levels (summer and winter) (Table 2). Surprisingly, the control group had significantly increased their backward falling technique at follow-up. The exercise group showed significant improvements in all three falling techniques (backward, forward, and sideways) at the 20-month follow-up (Table 2), and statistically significant differences were detected between the groups for fall competence forward (*p* = 0.033) and fall competence to the side (*p* = 0.009).

After the second full 12-week intervention, both groups showed significant improvements in scores of the SPPB, Mini-BESTest, and all falling techniques (backward, forward, and sideways) (Table 3). The between-group analysis revealed a significant difference only in the Mini-BESTest score between the groups at the 12-week follow-up (*p* = 0.023); however, both groups showed significant improvements at the 12-week follow-up in the within-group analysis (Table 3). The previous control group had a significant improvement in decreased pain (*p* = 0.005) and increased health condition (*p* = 0.037), but similar changes were not seen in the exercise group. The exercise group showed a significant improvement in activity level (summer) (*p* = 0.022) and fall-related self-efficacy (*p* = 0.049); similar changes were not seen in the previous control group. No adverse effects of the intervention were observed.

## 4. Discussion

This RCT investigated the effects of a group-based, multicomponent exercise program, including high-challenge strength and balance exercises, as well as teaching falling techniques. The intervention was effective in increasing physical function (strength and balance) and falling techniques in both groups. One of the most clinically relevant results judged by the research team was related to learning the falling techniques. First, the results indicated that motor skills and safe landing strategies can be learned in a short period of time (6–9 weeks) and that these skills were maintained over time (20 months). Second, both groups significantly increased their falling techniques after the 12-week intervention, which strengthens the previous result from the prior 6–9 weeks of intervention. These results address previous discussions that older adults may need a longer period of practice of new movements and motor skills needed to learn falling techniques [25], which was not confirmed by our study results. From a real-life perspective, this study investigates falling techniques from a learning aspect in a specific setting; it is necessary to verify the effectiveness and suitability of the learned strategies in real-life settings and in real-life falls [26]. Future studies need to focus on the possible effects of different falling techniques since hip impact from falls has been shown to have a higher impact when falling forward or sideways than when falling backward [27]. As a way to address falling sideways, the tuck-and-roll strategy has been shown to reduce fall impact severity [28], and our results confirm that these suggested falling techniques (forward and sideways) are trainable for older adults.

The Judo4Balance program was effective in increasing physical performance, including strength, balance, and learning falling techniques. These results add to the body of literature that 1 h of exercise for 12 weeks (12 sessions) with a high-challenge group-based program increased important physical functions related to reducing falls. These results present less volume than previous research suggests [6,29] in fall prevention and provide valuable knowledge when designing fall prevention programs. The results of this study may be of high clinical relevance because fall prevention programs fail to be effective when fidelity is neglected, such as limited time for professionals, lack of progressively challenging exercises, adherence to the exercise, and social context [6,30,31,32]. This study addresses several of these known shortcomings, including social context (group-based), highly progressively challenging exercises led by a professional trainer, and one session per week for 12 weeks.

One interesting finding from the follow-up at 20 months was the significant decrease in physical activity in the control group. Since sedentary behavior is a common risk factor that results in a decline in physical function [33,34], isolation during the pandemic most certainly negatively affects older adults’ social lives, leading to a higher risk of loneliness and depression [35], which physical activity and exercise can counteract.

Furthermore, participation in social activities and group-based exercises are considered important for the health of older adults [36]. In addition, group-based fall preventive exercise is favored over individually performed exercise [37], and highly challenging exercise programs are recommended and warranted.

Physical measures in this study, the SPPB and Mini-BESTest, revealed significant improvement at follow-up, despite the fact that this older adult group was, in general, a highly functional group, with a median score of 11/12 on the SPPB and 21/28 on the Mini-BESTest at baseline. The normative values of these tests in the same age group are suggested as follows: for the Mini-BESTest, a score of ≤21 differentiates those with and without postural response deficits with a sensitivity of 89% and specificity of 81% [38]. For the SPPB, a score of 10–12 has been suggested as a normative score for highly functional older adults [39]. Some discussions were held among the research team about the significant differences between the groups on balance performance at the last follow-up after the 12-week intervention. One explanation was related to detraining and retraining, leading to the benefits for the Judo4Balance group to regain the post-training level condition compared to the control group.

The strength of this study compared to many previous investigations is the interventions design that combines physical training and falling techniques. Emerging evidence indicates that acute bouts of physical exercise promote motor memory consolidation [40]; as a result, learning of falling techniques will be facilitated. On the other hand, improved fall strategies enable more rapid progression in physical training. Thus, this specific combination makes the intervention more efficient and less time-consuming compared to other fall prevention programs.

On a general level, the results of the study provide an important insight into the preservation of motor learning ability in older adults. Furthermore, specific motor skills related to fall strategies appear to be long lasting in this age group. The next important challenge is to establish whether improved physical status combined with enhanced falling techniques will reduce falls and fall-related injuries in long-term follow-ups. In addition, it will be important to establish whether enhanced physical status and improved fall strategies contribute to independence and long-term life satisfaction. Previous studies have shown that older adults with a high level of physical activity have more functional skills and fewer difficulties performing the activities of daily living and that they value their autonomy and health better [41,42].

On a practical level, the results indicate that relatively short training intervention designed with the specific aim of reducing the risk for falls and fall-related injuries is safe and effective and generates long-lasting effects in the older population. Thereby, the study indicates that this could be a scalable, cost- and time-effective answer to a well-known threat to older adults’ health.

### Limitations

This RCT deviated from the original protocol owing to the COVID-19 pandemic beginning in March 2020. The restart of the intervention consisted of 79 participants with pre-post measurements (37 from the intervention group and 42 from the control group). The restart included both groups, and this should also be considered a limitation; therefore, the results should be interpreted with caution. However, since we obtained significant results, we believe that the exercise was effectively evaluated by the chosen measurements in this study.

## 5. Conclusions

High-challenge multicomponent exercise programs, including learning falling techniques, appear to be effective in increasing physical function among older adults. Further, learning falling techniques in 6–9 weeks may lead to partially sustained fall competence. Thus, teaching a safe landing strategy may offer a complementary approach to fall prevention programs aimed at reducing fall-related injuries. If effective, safe landing strategies offer the potential to reduce fall-related injuries and maintain functional independence in older adults and need to be further investigated in future studies.

## Figures and Tables

**Table 1:** Baseline characteristics for all participants at baseline (*n* = 199).

Baseline Characteristic	Total	Exercise Group, Median (Min–Max)	Control Group, Median (Min–Max)
Sex (female/male)	79%/21% (*n* = 199)	76%/24%	82%/18%
Age (mean, SD)	72 (4, 9) (*n* = 199)	72 (4.5)	73 (5.2)
BMI >25 kg/m^2^	52% (*n* = 196)	54%	50%
EQ-5D-3L score, mobility (1–3)	3 (2–3) (*n* = 197)	3 (2–3)	3 (2–3)
EQ-5D-3L score, self-care (1–3)	3 (1–3) (*n* = 197)	3 (1–3)	3 (1–3)
EQ-5D-3L score, activity (1–3)	3 (2–3) (*n* = 197)	3 (2–3)	3 (2–3)
EQ-5D-3L score, pain (1–3)	2 (1–3) (*n* = 197)	2 (1–3	2 (1–3)
EQ-5D-3L score, anxiety (1–3)	3 (1–3) (*n* = 196)	3 (1–3)	3 (2–3)
Rated health status (0–100)	80 (25–100) (*n* = 195)	80 (25–100)	80 (36–100)
Activity level, summer (1–6)	4 (1–6) (*n* = 197)	4 (2–6)	4 (1–6)
Activity level, winter (1–6)	4 (1–6) (*n* = 198)	4 (1–6)	4 (1–6)
FES-S score (0–130)	127 (52–130) (*n* = 196)	126.5 (52–130)	127 (65–130)
SPPB score (0–12)	11 (2–12) (*n* = 198)	11 (2–12)	11 (5–12)
Mini-BESTest score (0–28)	22 (4–27) (*n* = 198)	22 (11–27)	21 (4–27)
Fall competence backward score (0–4)	1 (0–4) (*n* = 198)	1 (0–3)	1 (0–4)
Fall competence forward score (0–4)	1 (0–4) (*n* = 197)	1 (0–3)	1 (0–4)
Fall competence sideways score (0–4)	1 (0–4) (*n* = 196)	1 (0–4)	1 (0–4)

SD, standard deviation; BMI, body mass index; EQ-5D, EuroQoL-5D-3L; FES-S, Falls Self-Efficacy Scale Swedish version; SPPB, Short Physical Performance Battery.

**Table 2:** Descriptive statistics at the 20-month follow-up and within group analysis.

Outcome	Exercise Group	*n*	*p*-Value	Control Group	*n*	*p*-Value
EQ-5D-3L score, mobility (1–3)	3 (2–3)	37	0.414	3 (2–3)	41	0.564
EQ-5D-3L score, self-care (1–3)	3 (3–3)	37	0.317	3 (3–3)	41	0.180
EQ-5D-3L score, activities (1–3)	3 (2–3)	37	0.564	3 (3–3)	41	1.0
EQ-5D-3L score, pain (1–3)	2 (1–3)	37	0.366	3 (1–3)	41	0.197
EQ-5D-3L score, anxiety (1–3)	3 (2–3)	37	1.0	3 (1–3)	41	0.593
Rated health status (0–100)	75 (50–99)	37	0.852	80 (40–100)	41	0.058
Activity level, summer (1–6)	4 (2–6)	37	0.140	4 (1–6)	41	0.002
Activity level, winter (1–6)	4 (2–6)	37	0.870	4 (1–6)	42	0.003
FES-S score (0–130)	125 (66–130)	37	0.284	124 (66–130)	41	0.563
SPPB score (0–12)	12 (5–12)	37	0.463	11 (6–13)	42	0.248
Mini-BESTest score (0–28)	22 (6–27)	37	0.696	21 (7–27)	42	0.830
Fall competence backward score (0–4)	1 (0–4)	37	0.011	1 (0–4)	42	0.026
Fall competence forward score (0–4)	1 (0–4)	37	0.029	1 (0–3)	42	0.527
Fall competence sideways score (0–4)	1 (0–4)	37	0.010	1 (1–4)	42	0.167

EQ-5D, EuroQoL-5D-3L; FES-S, Falls Self-Efficacy Scale Swedish version; SPPB, short physical performance battery.

**Table 3:** Descriptive statistics and pre-post 12-week intervention within group analyses.

Measurement	Exercise Group	*n*	*p*-Value	Control Group	*n*	*p*-Value
EQ-5D-3L score, mobility (1–3)	3 (2–3)	30	0.157	3 (2–3)	28	0.564
EQ-5D-3L score, self-care (1–3)	3 (3–3)	30	1.0	3 (3–3)	28	1.0
EQ-5D-3L score, activity (1–3)	3 (2–3)	30	0.564	3 (3–3)	28	1.0
EQ-5D-3L score, pain (1–3)	2 (1–3)	30	0.180	2 (1–3)	27	0.005
EQ-5D-3L score, anxiety (1–3)	3 (2–3)	30	0.705	3 (2–3)	28	0.414
Rated health status (0–100)	80 (50–98)	30	0.089	80 (50–100)	28	0.037
Activity level, summer (1–6)	4 (3–6)	31	0.022	4 (2–6)	31	0.253
Activity level, winter (1–6)	4 (3–6)	31	0.380	4 (2–6)	31	0.490
FES-S score (0–130)	128 (86–130)	21	0.049	127.5 (8–130)	24	0.368
SPPB score (0–12)	12 (10–12)	31	0.009	12 (8–12)	31	<0.001
Mini-BESTest score (0–28)	25 (18–28)	31	<0.001	24 (18–28)	31	<0.001
Fall competence backward score (0–4)	4 (1–4)	31	<0.001	4 (1–4)	31	<0.001
Fall competence forward score (0–4)	3 (1–4)	31	<0.001	3 (1–4)	31	<0.001
Fall competence sideways score (0–4)	3 (1–4)	28	<0.001	3 (1–4)	25	<0.001

EQ-5D, EuroQoL-5D-3L; FES-S, Falls Self-Efficacy Scale Swedish version; SPPB, Short Physical Performance Battery.

## Data Availability

The data presented in this study are available upon request from the corresponding author. The data are not publicly available because of privacy or ethical restrictions.
